# A Case Report of a Co-Amoxiclav-Induced Black Hairy Tongue

**DOI:** 10.7759/cureus.58657

**Published:** 2024-04-20

**Authors:** Abobakr Fahdawi, Zay Yar Aung, Muhammad Samar Iqbal

**Affiliations:** 1 Acute Internal Medicine, Northampton General Hospital, Northampton, GBR; 2 Geriatrics, Royal Bolton Hospital, Bolton, GBR

**Keywords:** antibiotic treatment, medications, lingua villosa nigrais, co-amoxiclav, black hairy tongue

## Abstract

A black hairy tongue is a benign, self-limiting condition characterized by the discolouration of the tongue due to defective desquamation. Clinical presentation varies, with most cases being asymptomatic although aesthetically unpleasant to the patient. Prevalence varies geographically, ranging from 0.6% to 11.3%. It can be triggered by various factors such as medications, smoking, alcohol, poor oral hygiene, or even underlying systemic conditions such as malignancy. Several antibiotics such as doxycycline, erythromycin, amoxicillin-clavulanate, metronidazole, and piperacillin-tazobactam, have been reported to cause black hairy tongues. Onset can range from a few weeks to as long as five weeks. Diagnosis relies on clinical assessment with a good history and visual examination. Definitive treatment remains unclear, but the condition typically improves by identifying and discontinuing the causative agent and maintaining adequate oral hygiene. Complications are rare, and the prognosis is excellent. This case report aims to raise awareness of the association between the black hairy tongue and co-amoxiclav, which may impose additional burdens on patients, healthcare providers, and the health system if failed to be recognized and treated appropriately.

## Introduction

A black hairy tongue (BHT), also known as a lingua villosa nigra, is a rare, benign, and self-resolving condition characterized by black discolouration and a hair-like appearance on the dorsal surface of the tongue [1]. Its exact mechanism remains unclear, but BHTs may be attributed to either environmental (poor hygiene, foods, etc.) or intrinsic factors like delayed, inadequate desquamation of filiform papillae [2]. Prevalence varies geographically, ranging from 0.6% to 11.3% [3-5]. The onset can occur within a few weeks or extend up to five months [2].

Co-amoxiclav, a combination of amoxicillin (a β-lactam antibiotic) and potassium clavulanate (a β-lactamase inhibitor) is commonly used to treat various bacterial infections including chest and urinary tract infections. It can be administered orally or intravenously [6,7]. The British National Formulary (BNF) lists BHTs as potential side-effects of oral co-amoxiclav with an unknown frequency [7]. The association between co-amoxiclav and black tongue has been reported in both pediatric and adult populations [8-10]. Antimicrobial use may alter the normal oral commensals and may discolour the tongue. *Porphyromonas gingivalis*, a Gram-negative anaerobic rod bacteria, and Candida species may be associated with tongue discolouration [10,11].

Our patient developed a black hairy tongue after the use of co-amoxiclav for five days, and it resolved with conservative management of simple oral hygiene. The case report highlights the uncommon adverse effects of co-amoxiclav with probable adverse reaction on the Naranjo score [12].

## Case presentation

**Figure 1:**
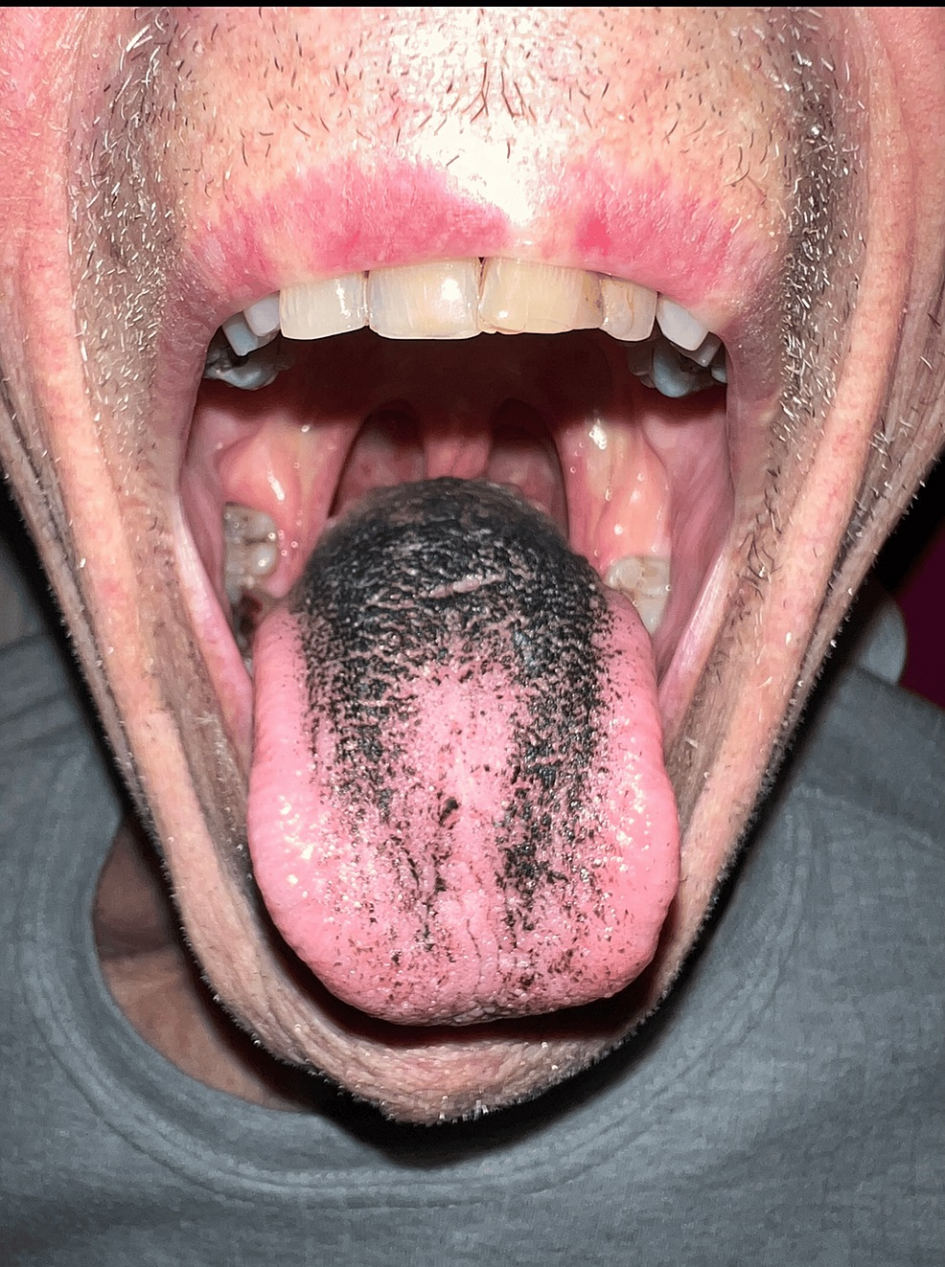
Black hairy tongue at first presentation

**Figure 2:**
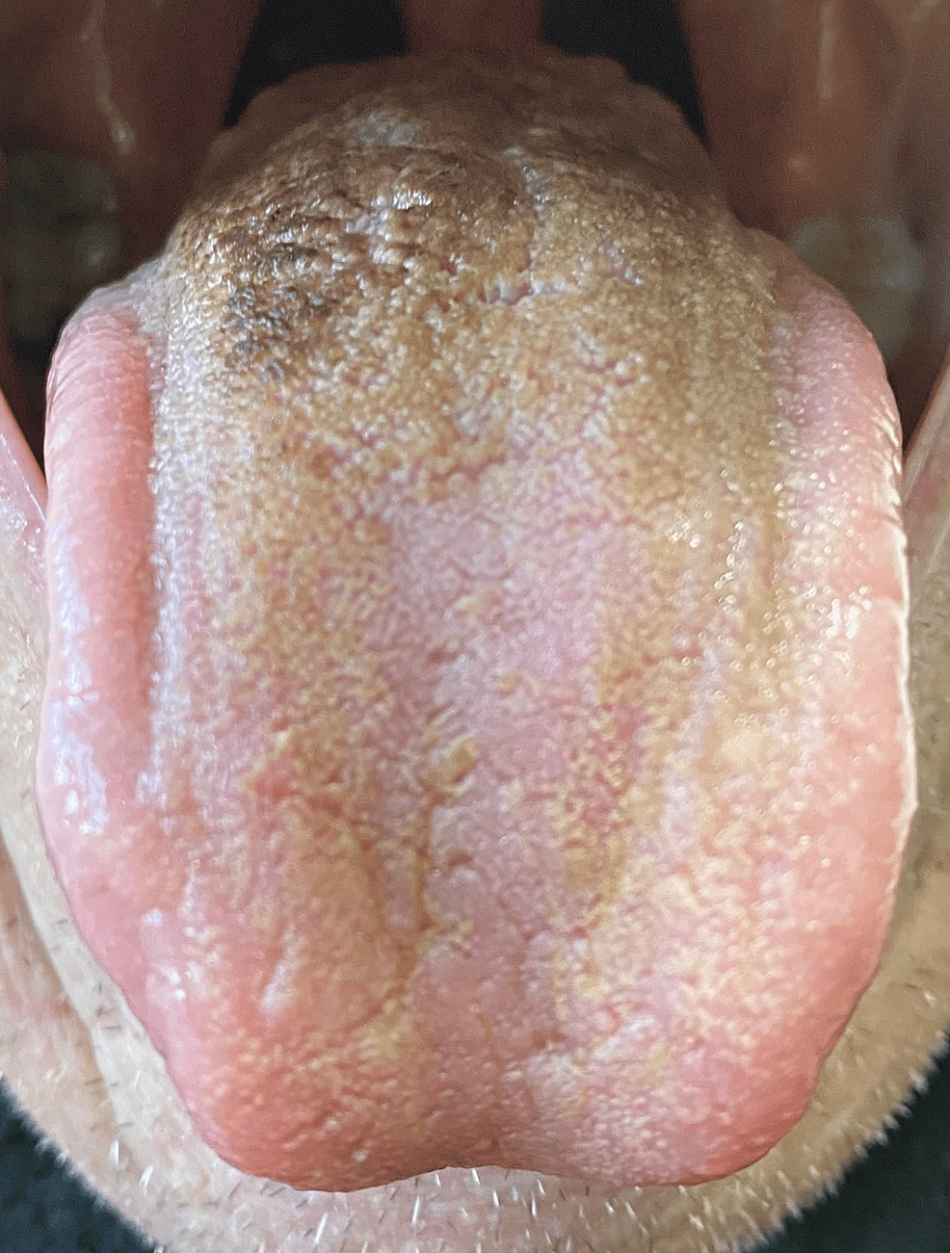
Resolution of the discolouration after three weeks with conservative management

An elderly gentleman in his 80s, admitted for community-acquired pneumonia, developed a black coating on his tongue. His respiratory symptoms were improving with two days of intravenous co-amoxiclav 1.2 gram for two days followed by an oral form of 625 mg three times daily. However, he noticed his tongue had turned black on the fifth day of the treatment regime. He denied having tongue pain, bleeding, ulcers, or any loss of weight/appetite. Additionally, he had not consumed black-coloured food or drinks. He is a non-alcoholic and is an ex-smoker of three decades. His regular medications included ramipril, atorvastatin, aspirin, and bisoprolol with no recent changes except for co-amoxiclav for his pneumonia treatment. On physical examination, the dorsum part of the tongue, particularly the filiform, foliate, and vallate papillae, appeared black whilst the buccal mucosa was normal (Figure 1). There were no signs of lymphadenopathy, rash, or joint swelling. Full blood count, renal function, liver function, thyroid function, and viral screening were all within normal range. The Naranjo score [12], a widely used scoring system for adverse drug reactions, was 5 indicating probable adverse reaction. Consequently, we concluded that the most probable diagnosis was a black hairy tongue secondary to co-amoxiclav, given the absence of red flag clinical features or other risk factors in clinical history and physical examination. The patient was reassured and advised to complete the seven-day course of co-amoxiclav while maintaining good oral hygiene practices with regular brushing of the tongue, gargling, and adequate hydration. His tongue discolouration improved significantly after 10 days and returned to almost normal after three weeks, as depicted in Figure 2. 

## Discussion

We highlight the association between black hairy tongues and co-amoxiclav. The clinical presentation of black hairy tongues varies among patients with many being asymptomatic, though the condition is aesthetically unpleasant. No objective diagnostic criteria have yet been established for BHT [10]. Hence, diagnosis typically requires a detailed history and visual examination of the tongue. Proposed predisposing factors include smoking, poor oral hygiene, excess coffee/tea consumption, alcohol, and certain medications, including antibiotics, malignancy, and immunosuppressed conditions [5]. Differential diagnoses include acanthosis nigiricans, oral hairy leukoplakia, pigmented fungiform papillae, Addison's disease, and black staining from food colourings [13]. 

Certain antibiotics such as minocycline, doxycycline, erythromycin, linezolid, amoxicillin, clavulanate, metronidazole and piperacillin-tazobactam have been implicated in altering the filiform papillae of the tongue leading to the formation of elongated ‘hairs’ due to the defective desquamation of the cells [10]. Liccioli et al. proved the occurrence of black tongues due to a positive hypersensitivity reaction (lymphocyte transformation test) to co-amoxiclav in paediatric cases [8]. 

Our patient exhibited a black hairy tongue with no identifiable risk factors except co-amoxiclav use for his pneumonia. Nonetheless, the Naranjo score [12] of 5 indicates a probable adverse reaction supported by previous conclusive reports, the appearance of adverse events after the suspected drugs and ultimately the improvement in the reaction when the affecting drug was discontinued. 

The prognosis is generally good as the condition is self-limiting and when following the general advice of good oral hygiene and staying away from triggering factors such as smoking, alcohol, and caffeine. Good patient counselling is important to avoid patient anxiety [5]. Our patient’s black tongue resolved within three weeks with good oral hygiene and conservative management. However, a case-series study by Ren et al. researched that microbiologic culture of the tongue grew fungi in three patients [10]. In the case report of Sheikh et al., the patient was treated with oral fluconazole because the tongue scrapings confirmed Candida infection and the black tongue resolved after seven days with fluconazole [9]. Therefore, we should consider for tongue swab if there are clinical features of sinister causes or if the black tongue is not resolved with conservative management.

We highlight an uncommon adverse effect of co-amoxiclav despite no long-term complications. Furthermore, the co-amoxiclav-induced black hairy tongue may prompt unwarranted investigations due to patient and healthcare professional anxiety leading to the depletion of valuable resources.

## Conclusions

While antibiotic-induced black hairy tongues are not uncommon, they can provoke anxiety for both patients and healthcare professionals. Therefore, a comprehensive clinical history, examination, and medication review are essential, as causes could range from simple poor oral hygiene to more sinister conditions such as cancers and immunocompromised cases like HIV. The management of an antibiotic-induced black hairy tongue primarily involves reassurance, promoting good oral hygiene, and regular follow-up to address this mentally stressful condition, thus avoiding unnecessary expending of resources.
